# Prediction of *In*-*silico* ADME Properties of 1,2-O-Isopropylidene Aldohexose Derivatives 

**Published:** 2014

**Authors:** Strahinja Z Kovačević, Lidija R Jevrić, Sanja O Podunavac Kuzmanović, Eva S Lončar

**Affiliations:** a *University of Novi Sad, Faculty of Technology, Department of Applied and Engineering Chemistry, Bulevar cara Lazara 1, 21000 Novi Sad, Serbia. *

**Keywords:** 1, 2-*O*-isopropylidene derivatives of aldohexoses, *In-silico*, ADME, PCA, Polynomial regression

## Abstract

Retention behaviour of molecules mostly depends on their chemical structure. Retention data of biologically active molecules could be an indirect relationship between their structure and biological or pharmacological activity, since the molecular structure affects their behaviour in all pharmacokinetic stages. In the present paper, retention parameters (*R*_M_^0^) of biologically active 1,2-*O*-isopropylidene aldohexose derivatives, obtained by normal-phase thin-layer chromatography (NP TLC), were correlated with selected physicochemical properties relevant to pharmacokinetics*, i.e*. absorption, distribution, metabolism, and elimination (ADME) properties. Conducted correlation analysis showed high dependence between *R*_M_^0^ and blood brain barrier penetration, skin permeability, enzyme inhibition, binding affinity to nuclear receptor ligand and G protein-coupled receptors, as well as lipophilicity (expressed as Hansh-Leo’s parameter *C*log *P*). The statistical validity of the established polynomial dependence of the second degree between *R*_M_^0^ and mentioned ADME properties was confirmed by standard statistical measures and leave-one-out *cross*-validation method. On the basis of *in-silico *calculated ADME properties and retention data, the similarity between studied molecules was examined using principal component analysis (PCA). The obtained results indicate the possibility of predicting ADME properties of studied compounds on the basis of their retention data (*R*_M_^0^). These preliminary results could be treated as guideline for selecting new 1,2-*O*-isopropylidene aldohexose derivatives as drug candidates.

## Introduction

The biological role of aldohexoses (*i.e*. glucose, mannose, galactose, *etc*.) is well known. Therefore, in several scientific publications attention was paid to the investigation of biological and pharmacological activity of some aldohexose derivatives, such as isopropylidene derivatives. In earlier studies it was found that isopropylidene derivatives of aldohexoses exhibit immunomodulatory function (1) and interaction with the human erythrocyte glucose transport system (2). Recent researches indicate that these compounds can induce erythroid differentiation of human leukemic K562 cells (3-5), and manifest antimicrobial activity (6, 7). Besides, mentioned derivatives have conveniently been used as starting compounds and key intermediates in the synthesis of several biologically active compounds (8-11). The compounds studied in this paper may exhibit pharmacological activity based on their structural similarity to the known, active compounds. The potential use of these derivatives as therapeutic agents mostly depends on their pharmacokinetics and pharmacodynamics. Pharmacokinetic phase includes absorption, distribution, metabolism, and elimination (ADME) of the drug. Screening and optimizing ADME properties in the early stage of the drug development process are widely accepted (12). Fast evaluation of ADME properties will save both time and expense. However, due to the complex nature of these properties and the time-consuming experimental procedures involved, these properties are not apt to experimental screening (13). Therefore, a large number of *in-silico *ADME models have been developed (14, 15). According to the analysis of the failed new chemical entities, the leading causes of failures (~50–60%) are poor ADME properties and adverse effects, which contribute significantly more than a lack of efficacy (~30%) (16). 

Many of the factors that influence drug action apply to all aspects of the pharmacokinetic phase. Molecular structure is an important factor for ADME properties of investigated molecules, and can be used as a predictor of their pharmacokinetics. Since the retention behaviour mostly depends on molecular structure, in the present study correlation between retention data and several ADME properties of 1,2-*O*-isopropylidene derivatives of aldohexoses was examined using chemometric approach. Retention behaviour of studied derivatives was examined using NP TLC, and it is described by the *R*_M_ value defined by the Bate-Smith equation (17, 18):

Equation (1) $$R_{M}=\log[(1/R_{f})–1]$$

Where *R*_f _is the so-called retardation factor, defined as the ratio of the single zone distance and the solvent front. The value of *R*_M_ depends linearly on the logarithm of the concentration of the organic modifier in the mobile phase (φ) according to the following equation: 

Equation (2)

$R_{M}={R_{M}}^{0}+S\mathrm{.\varphi}$

Where *R*_M_^0^ is the intercept and *S *is the slope. In this paper, *R*_M_^0 ^ factors were correlated with *in-silico *ADME properties of 1,2-*O*-isopropylidene derivatives of aldohexoses. 

The main purpose of the conducted correlation analysis was to determine the ability to predict ADME properties of these molecules using chromatographic retention data, since the chromatography has been shown to be quite successful in modeling physicochemical and biological properties (19-22). Due to presence of relatively large number of variables for each compound, it was useful to apply PCA on calculated ADME properties and *R*_M_^0^ values in order to reveal some similarities among studied compounds. 

## Experimental

*Studied compounds*

**Figure 1:**
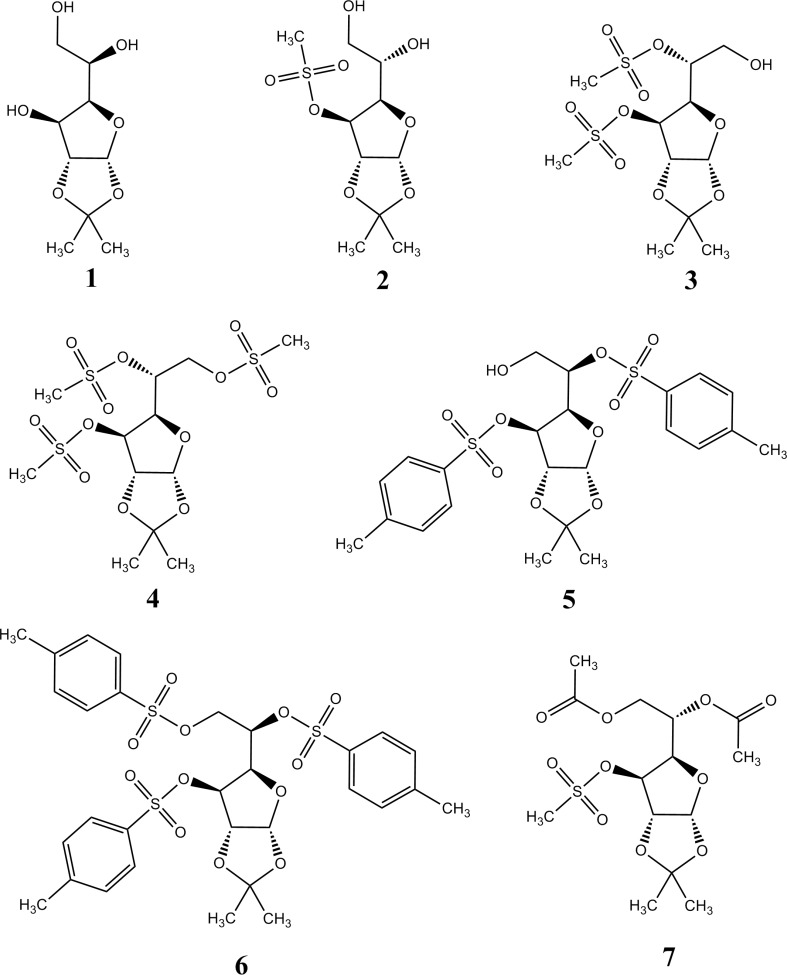
Structural formulas of the examined molecules

The structures of the aldohexose derivatives examined in this paper are presented in Figure 1, and their names are shown in Table 1. These compounds contain several functional groups which differ in polarity: hydroxyl, methanesulfonyl, *p*-toluenesulfonyl, and acetyl group. 

**Table 1:** The names of the examined molecules

**Molecule **	**Name **
1	1,2-*O*-isopropylidene-α-D-glucofuranose
2	1,2-*O*-isopropylidene-3-*O*-methanesulfonyl-β-L-idofuranose
3	1,2-*O*-isopropylidene-3,5-di-*O*-methanesulfonyl-α-D-glucofuranose
4	1,2-*O*-isopropylidene-3,5,6-tri-*O*-methanesulfonyl-α-D-glucofuranose
5	1,2-*O*-isopropylidene-3,5-di-*O*-*p*-toluenesulfonyl-α-D-glucofuranose
6	1,2-*O*-isopropylidene-3,5,6-tri-*O*-*p*-toluenesulfonyl-α-D-glucofuranose
7	5,6-di-*O*-acetyl-1,2-*O*-isopropylidene-3-*O*-methanesulfonyl-β-L-idofuranose

*Thin-Layer Chromatography (TLC)*

Analytical procedure for TLC was described in detail previously (23). *R*_M_^0^ factors obtained using three different mobile phases (cyclohexane as a diluent; acetone, dioxane, tetrahydrofuran as modifiers) were included in the present study. Data for linear correlation between *R*_M_ and φ were previously reported (23).

*Calculation of ADME properties*

On the basis of 2D structural models, drawn in ChemBioDraw Ultra version 12.0 software (Cambridge Software), ADME properties of studied compounds were calculated using online PreADMET program and Molinspiration program (24-26). The values of the observed properties are presented in Table 2.

**Table 2:** The values of ADME properties of the studied compounds obtained using *in-silico *method.

**Molecule:**	**1**	**2**	**3**	**4**	**5**	**6**	**7**
PPB%	11.555	19.053	46.565	73.918	100.000	100.000	36.710
BBB (C_brain_/C_blood_)	0.284	0.076	0.052	0.052	0.127	0.159	0.062
HIA%	57.079	59.210	61.849	65.037	96.609	99.630	69.975
Caco-2 (nm/sec)	0.184	1.295	1.361	1.000	7.592	12.934	1.541
MDCK (nm/sec)	4.087	2.018	1.433	0.929	0.044	0.043	5.049
SP (log*K*_p_)	-5.284	-3.983	-2.076	-1.585	-1.086	-0.796	-2.279
GPRC	-0.84	-0.56	-0.34	-0.41	-0.22	-0.41	-0.41
EI	0.34	0.63	0.53	0.47	0.24	-0.20	0.44
ICM	-0.70	-0.83	-0.69	-0.68	-0.49	-1.13	-0.67
KI	-1.16	-0.88	-0.54	-0.52	-0.42	-0.79	-0.66
NRL	-1.06	-0.45	-0.19	-0.26	-0.27	-0.66	-0.26
PI	-0.84	-0.17	-0.01	-0.02	-0.01	-0.06	-0.05
*C*log *P*	-0.980	-1.130	-0.620	-0.210	2.860	4.999	0.750

Generally, only the unbound drug molecule is available for diffusion or transport across cell membranes and for interaction with a pharmacological target. As a result, a degree of plasma protein binding (PPB%) of a drug influences on the drug’s action, its disposition and efficacy. Therefore, the PPB% is an important pharmacokinetic factor and is determinant in the actual dosage regimen (frequency), but not important for the daily dose size (27). 

Blood-brain barrier (BBB) penetration is crucial in pharmaceutical sphere because CNS-active compounds must pass through it. BBB penetration is presented as concentration ratio of steady-state of radiolabeled compounds in brain (C_brain_) and peripheral blood (C_blood_).

Predicting human intestinal absorption (HIA%) of drugs is very important for identifying potential drug candidate. HIA% data are the sum of bioavailability and absorption evaluated from ratio of excretion or cumulative excretion in urine, bile and feces (28).

For the development of bioactive molecules as therapeutic agents, oral bioavailability is often an important consideration. Caco-2 cell model and Madin-Darby canine kidney (MDCK) cell model have been recommended as a reliable *in-vitro *model for the prediction of oral drug absorption. Caco-2 cells, a well-differentiated intestinal cell line derived from human colorectal carcinoma, display many of the morphological and functional properties of the *in-vivo *intestinal epithelial cell barrier (29). Advantage of MDCK cells is that its growth period is shorter than Caco-2 cell, so MDCK cells system may be used as good tool for rapid permeability screening (30). 

In the pharmaceutical, cosmetics and agrochemical fields, it is important to predict the skin permeability (SP) rate as a crucial parameter for the transdermal delivery of drugs. PreADMET program predicts *in-vitro *SP and the result value is given as log*K*_p_. *K*_p_ (cm/h) is defined as (31):

Equation (3) $$K_{p}=(K_{m}\mathrm{.D})/h$$

where *K*_m_ is distribution coefficient between *stratum corneum *and vehicle, *D *is average diffusion coefficient (cm^2^/h), and *h *is thickness of skin (cm).

Calculation of bioactivity scores for G protein-coupled receptors ligand (GPCR), ion channel modulation (ICM), kinase inhibition (KI), nuclear receptor ligand (NRL), protease inhibition (PI), and enzyme inhibition (EI) was done using Molinspiration software. These values indicate binding affinity of examined compounds to the mentioned receptors and enzymes (negative values mean low affinity, while positive values indicate greater affinity). 

Lipophilicity of a compound is an important physicochemical parameter, which determines biological processes as it is related to absorption, bioavailability, hydrophobic drug-receptor interacions, metabolism, and toxicity (32). The lipophilicity affects the penetration of bioactive molecules through the apolar cell membrane, and it is a very important factor for pharmacokinetic phase (33). Hansh-Leo’s partition coefficient for *n*-octanol/water bi-phase system (*C*log *P*) was calculated using ChemBioDraw Ultra version 12.0 software.

*PCA*

PCA is a multivariate statistical method that is usually used to reduce the dimensionality (number of variables) of a large number of interrelated variables, while retaining as much of the information (variation) as possible. The first principal component (PC1) is chosen in the direction of the largest variance in the data set, followed by the second one that encloses the rest of the variability and so on (32). The corresponding loadings plot displays relationships between variables and can be used to identify variables (*R*_M_^0^ values and ADME properties in this study) that contribute to the positioning of the compounds on the scores plot and hence influence any observed groups in the data set. In this study PCA was carried out using Statistica 8 software (34).

*Correlation analysis and model validation*

The software package used for correlation analysis and model validation was NCSS 2007 and GESS 2006 (35). In the present study correlations between retention data (*R*_M_^0^) and presented ADME properties of examined compounds were examined.

Statistical validity of the established mathematical models was determined by statistical measures: Pearson’s correlation coefficient (*r*), standard deviation (*s*), and Fisher’s value (*F*). Predictive power of the mentioned models was tested by leave-one-out *cross*-validation method and validated by the calculation of the following parameters: *cross*-validated coefficient of determination (*r*^2^_CV_), adjusted coefficient of determination (*r*^2^_adj_), predicted residual sum of squares (*PRESS*), total sum of squares (*TSS*), and standard deviation based on predicted residual sum of squares (*S*_PRESS_) (36, 37). Optimal values of these parameters (*r*^2^ > 0.6, *r*^2^_CV_ > 0.5, *r*^2^_adj_ > 0.5, *F > F*_crit_., *PRESS *value lower than *TSS*, low values of *s *and *S*_PRESS_) indicate that the established mathematical models are statistically significant (36, 38).

## Results and Discussion

Retention behaviour of the examined 1,2-*O*-isopropylidene aldohexose derivatives was explained in detail in literature (23). Therefore, in this study the focus was on comparison of their retention and ADME properties. 

**Figure 2:**
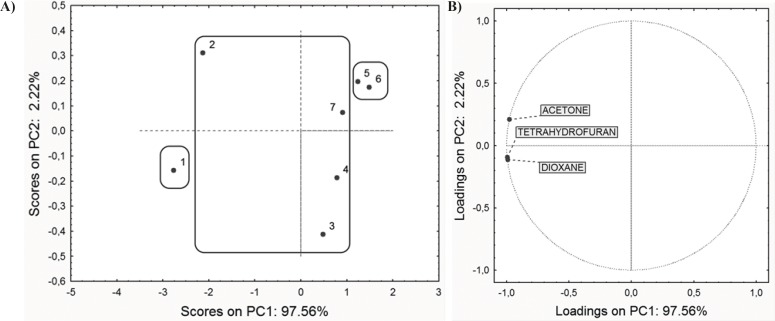
PC1-PC2 score plot (A) and factor loadings (B).

In the first step, PCA was performed on the retention data (*R*_M_^0^ values obtained for three chromatographic systems) and ADME properties in order to reveal similarities and dissimilarities among the studied compounds. PCA applied on the entire set of *R*_M_^0^ values resulted in a two-component model explaining 99.78% of the data variation (PC1 comprise 97.56% and PC2 2.22% of variances). The scores plot and the loadings plot of the first two PCs are presented in Figure 2.

Along the PC1 axis scores plot (Figure 2A) indicates that compounds 5 and 6 have the lowest retention factor, while the compound 1 has the highest retention, according to polarity of present substituents on C-3, C-5 and C-6 atoms (23). Substituents affect the polarity of compounds, and therefore affect the retention order in applied chromatographic systems. Loadings plot revealed that all applied modifiers have the highest negative impact to the PC1 (Figure 2B).

**Figure 3:**
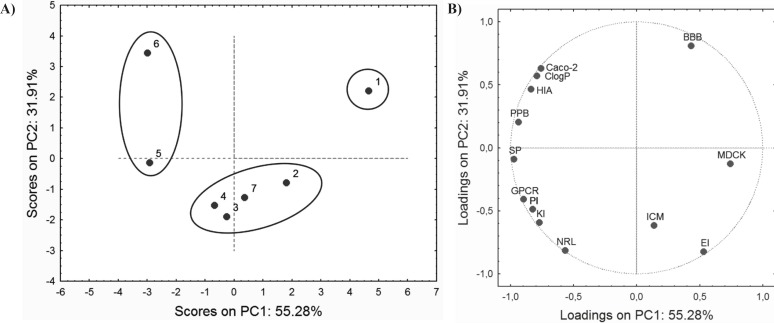
Score values (A) and factor loadings (B) of ADME properties for the first two PCs

The PCA performed on ADME properties resulted in a three-component model that explains 94.45% of total variance. The PC1 explains up to 55.28% of the variability, and the PC2 accounts for up to 31.91%. Score values and factor loadings of the first and the second PC are presented in Figure 3.

Loadings plot shows that the majority of ADME properties have a negative impact on PC1, while only BBB, ICM, EI and MDCK have a positive influence. As it can be observed from the score plot (Figure 3A), compounds are grouped in similar way as in score plot based on retention data (Figure 2A). This similarity may indicate connection between retention behaviour and ADME properties of the investigated compounds. 

Correlation analysis revealed that the relationship between some of calculated ADME properties and retention data obtained in chromatographic systems with dioxane and tetrahydrofuran as modifiers are best described with polynomial function of the second degree. It was found that retention factors (*R*_M_^0^) obtained in chromatographic system with aceton as modifier are not in good correlation with ADME properties. The statistical validity of the established models, as depicted in Table 3, was determined by *r*, *F*, and *s. *Correlation coefficient higher than 0.90 indicates very high correlation between *R*_M_^0^ and ADME properties. F*-*value is found statistically significant at 99% level since all the calculated F-values are higher as compared to tabulated values. 

**Table 3:** Correlations between retention data and ADME properties of studied derivatives

**Modifier**		**Dependent variable**	**Polynomial regression: Y ** ***= a · *** **(** *R*_M_^0^ **)** ^2^ ***+ b · ****R*_M_^0^ ***+ c***							**Eq** ***.***
		**Y**	***a***	***b***	***c***	***r***	***F***	***s***		
Dioxane	BBB		1.016	2.183	1.192	0.9662	28.14	0.02673	4	
Dioxane	SP		-1.318	-7.529	-9.142	0.9787	45.44	0.4093	5	
Dioxane	GPCR		-0.9401	-2.434	-1.919	0.9354	14.00	0.08551	6	
Dioxane	EI		-3.212	-6.094	-2.140	0.9686	30.36	0.08329	7	
Dioxane	NRL		-3.417	-7.508	-4.257	0.9735	36.27	0.08776	8	
Dioxane	*C*log *P*		20.12	35.50	13.39	0.9743	37.43	0.6351	9	
Tetrahydrofuran	BBB		0.7109	1.392	0.6833	0.9558	21.13	0.03051	10	
Tetrahydrofuran	SP		-0.7620	-5.146	-6.955	0.9733	36.01	0.4573	11	
Tetrahydrofuran	GPCR		-0.5468	-1.408	-1.253	0.9262	12.07	0.09124	12	
Tetrahydrofuran	NRL		-2.146	-4.374	-2.348	0.9241	11.69	0.1466	13	

Equations 4-13 were *cross*-validated by the leave-one-out method (Table 4). High values of *r*^2^_cv_ and *r*^2^_adj _(higher than 0.5) and *PRESS *values significantly less than *TSS *were obtained for all the models and indicate that these models have very good predictive power. 

**Table 4:** *Cross*-validation parameters for equations 4-13.

**Eq** ***.***	***r*** ^2^_cv_	***r*** ^2^_adj_	***PRESS***	***TSS***	***PRESS/TSS***	***S***_PRESS_
4	0.5158	0.9005	0.02085	0.04290	0.4860	0.05458
5	0.9058	0.9368	1.498	15.89	0.09427	0.4612
6	0.6613	0.8124	0.07921	0.2338	0.3388	0.1064
7	0.7546	0.9073	0.1100	0.4480	0.2455	0.1254
8	0.8254	0.9216	0.1027	0.5884	0.1745	0.1211
9	0.6165	0.9239	12.20	31.81	0.3835	1.320
10	0.5187	0.8703	0.02077	0.04290	0.4841	0.05447
11	0.8689	0.9211	2.083	15.89	0.1310	0.5455
12	0.5070	0.7867	0.1152	0.2338	0.4927	0.1283
13	0.5611	0.7809	0.2582	0.5884	0.4388	0.1921

The best correlations between *R*_M_^0^ and BBB, SP, GPCR, EI, NRL, and *C*log *P *parameters were obtained in chromatographic systems with dioxane and tetrahydrofuran as modifiers. Established models, presented in Table 3, indicate that retention factor (*R*_M_^0^) of the examined molecules could be considered as a predictor of skin permeability rate, blood-barrier penetration, partition coefficient *C*log *P*, enzyme inhibition, and binding affinity to nuclear receptor and G protein-coupled receptor. 

## Conclusion

Because of limited number of the molecules studied in the present paper, the presented results should be treated as very preliminary ones, but some conclusions could be drawn.

According to calculated ADME properties, examined molecules exhibit enzyme inhibition, but have less emphasized binding affinity to NRL and GPCR. In the present study it is shown that compounds which have high lipophilicity, also have lower retention and higher BBB permeability and SP rate. It was found that experimentally determined retention parameter (*R*_M_^0^) of studied 1,2-*O*-isopropylidene derivatives of aldohexoses were reliably correlated with *in-silico *calculated BBB penetration, SP rate, bioactivity score for EI, and binding affinity to NRL and GPCR, as well as partition coefficient for *n*-octanol/water bi-phase system (*C*log *P*). Standard statistical measures and *cross*-validation parameters indicate that the established mathematical dependences between retention parametres and ADME properties are statistically valid. Also, PCA applied on both the retention parameters and calculated ADME properties showed similar grouping of molecules. That could indicate the similarity between retention and ADME properties of the examined molecules. On the basis of presented results it can be concluded that the retention parameters obtained by NP TLC could be successfully used for prediction of biological activity and some *in-silico *ADME properties of studied compounds.
